# TRANSPORTATION IS THE DRIVER TO VISIT: DOES COMMUNITY CONNECTEDNESS AFFECT DEPRESSION SYMPTOMS OF RESIDENTS?

**DOI:** 10.1093/geroni/igz038.1312

**Published:** 2019-11-08

**Authors:** Vivian J Miller

**Affiliations:** University of Texas at Arlington, Arlington, Texas, United States

## Abstract

A recent study found that lack of transportation access (or, transportation disadvantage), together with travel time, is a major barrier for community members to visit their loved ones residing in long-term care nursing homes (Miller, 2018). This transportation disadvantage not only may contribute to decreased visitation but also prohibits family from providing social support to residents, which is imperative for residents to maintain decreased symptoms of depression, a sense of belonging, and highest well-being. A sequential mixed-methodological study was conducted across 11 CMS-certifiable nursing homes in North Central Texas to examine this effect of transportation disadvantage of community members on depressive symptoms of their family in long term care (N=89 dyads). Findings from this study will be presented. Additionally, implications for social work, transportation planning, policymakers, and other key professions will be discussed.

